# Tracheoesophageal Fistula Caused by Tracheostomy in a Patient with Myasthenia Gravis after a Myasthenic Crisis

**DOI:** 10.3389/fneur.2017.00217

**Published:** 2017-05-19

**Authors:** Chen Jiaxin, Li Jingjing, Zhu Kai, Zhou Zhou, Liu Weibin, Wang Haiyan, Feng Huiyu

**Affiliations:** ^1^Department of Neurology, The First Affiliated Hospital of Sun Yat-sen University, Sun Yat-sen University, Guangzhou, China; ^2^Guangdong Provincial Key Laboratory for Diagnosis and Treatment of Major Neurological Disease, Guangzhou, China

**Keywords:** myasthenia gravis, myasthenic crisis, tracheostomy, complication, tracheoesophageal fistula

## Abstract

A 57-year-old woman with myasthenia gravis (MG), who had experienced a myasthenic crisis, complained of coughing while drinking. At first, this appeared to be a sequela of the myasthenic crisis. However, after further investigation, the problem was identified as a tracheoesophageal fistula, a complication of tracheostomy. Here, we describe this special case in the hope that we can improve diagnostic accuracy by providing a reminder for other physicians to consider the differences between MG and tracheoesophageal fistula. It is very important to pay more attention to such situations in clinical scenarios and administer the most appropriate treatment without delay.

## Introduction

Myasthenia gravis (MG) is an antibody-mediated, autoimmune, neurological disorder. The clinical features of this condition include fluctuating weakness and excessive fatigue in the skeletal muscles. Such weakness is aggravated by activity and is alleviated with rest. There is a predilection for the external ocular, masticatory, and facial muscles. During a myasthenic crisis, the pharyngeal, laryngeal, respiratory, and limb muscles may also be affected. As MG is systemic, two or more of these muscles may be affected at the same time, causing a more complex condition, and sometimes even masking other diseases. When the pharyngeal and laryngeal muscles are involved, MG can present as coughing while drinking, dysphagia, and dysarthria (1). Patients who have a tracheoesophageal fistula may cough when they are on a liquid or semiliquid diet because foreign matter can easily enter the trachea; this phenomenon is similar to that seen with MG.

## Background and Case Presentation

A 57-year-old woman presented with fluctuant ptosis and diplopia. She had been diagnosed with MG in 1980. The neostigmine test was positive. Single-fiber electromyography with repetitive nerve electrical stimulation showed decreasing amplitudes (left facial, right accessory, and right ulnar nerves) under both low- and high-frequency stimulation (15 and 32%, respectively). Single-fiber electromyography revealed that all 15 pairs of the right extensor digitorum communis showed a mean jitter of 69 µs. The patient’s acetylcholine receptor antibody titer was significantly increased, despite the fact that the patient was regularly treated with a cholinesterase inhibitor, which normally controlled her symptoms effectively.

This patient had a long, yet well-regulated, medical history. In 2001, chest computed tomography (CT) showed thymic hyperplasia. Following thymectomy, she was prescribed prednisone to maintain a better status. Because of long-term steroid therapy, the patient has suffered from steroid-induced diabetes since 2014. Instead of taking oral medication, she had been maintained on insulin injections since 2015. That same year, she was also diagnosed with hypothyrea and was prescribed Euthyrox (Levothyroxine Sodium Tablets, Merck KGaA, Darmstadt, Germany) to relieve the symptoms.

On the 6th of March 2016, the patient developed a fever and cough, but did not take this seriously. After 3 days of these symptoms, this condition developed into a myasthenic crisis. By this time, her pharyngeal, laryngeal, respiratory, and limb muscles were all affected. She presented at a local hospital with nasal-sounding speech, difficulty chewing, dysphagia, respiratory difficulties, and limb weakness, for which she received tracheal intubation and mechanical ventilation. Since long-term mechanical-assisted ventilation is known to cause extreme throat swelling, and other complications, we performed tracheostomy 10 days later. In the meantime, the patient was given intravenous injections of high-dose immunoglobulin, cholinesterase inhibitors, immunosuppressive agents, antimicrobial medications, and received nasogastric intubation. After 27 days of treatment, the tracheal intubation catheter and nasogastric tube were removed. The patient’s chewing, respiration, and speech had been restored well, but she still coughed when drinking and experienced dysphagia. The local hospital regarded this as a sequela of the myasthenic crisis and advised her to undergo swallowing rehabilitation training. She was thus discharged from the hospital on approximately 1 month after admission.

Four weeks later, the patient showed no significant improvement. Each time she tried to drink, it caused her to cough. This situation increased the risk of pulmonary infection and could have led to aspiration pneumonia. On the 6th of May 2016, she was seen by our Department of Neurology and was hospitalized. Blood tests showed an elevated white blood cell count (11.93 × 10^9^/L), a high ratio of neutrophilic granulocytes (0.771), a low hemoglobin concentration (96 g/L), and a lower-than-normal serum albumin level (29.0 g/L).

We used the Myasthenia Gravis Foundation of America (MGFA) Clinical Classification to grade the severity of the patient’s disease. When she first came to the hospital, her MGFA classification was IIIb (defined as “predominantly affecting oropharyngeal or respiratory muscles, or both; with lesser or equal involvement of limb or axial muscles, or both”). Her quantitative MG score was 12 (defined by the MGFA criteria as “swallowing 4 oz. of water causes severe coughing/choking or nasal regurgitation”).

To reduce the risk of aspiration, they underwent nasogastric tube placement. For such an extreme situation, the routine treatments for MG were prepared for protracted use (Table 1) (2, 3). Weekly records showed that the MGFA Clinical Classification remained unchanged and that the quantitative MG score had stabilized (4). The patient failed swallowing function evaluation tests four times (at the time of admission, and at each of three subsequent weekly evaluations); each time she tried to drink, she experienced a severe coughing episode. Unfortunately, the patient was no better after the 3 weeks of treatment.

**Table 1:** **The treatments for the patient during the repeated admission before the clarify diagnosis**.

Treatment	Dosage	Course (days)
Intravenous high-dose immunoglobulin	20.0 g i.v. drip q.d. 5.0 g i.v. drip q.d.	3 5
Cholinesterase inhibitors	60 mg q.i.d.	21
Immunosuppressive agents: tacrolimus	0.5 mg q.d. 1.0 mg q.d. 2.0 mg q.d.	9 7 5
Albumin infusion	20.0 g i.v. drip q.d. 5.0 g i.v. drip q.d.	3 8
Anti-infective medications	Cefatriaxone 1.0 g i.v. drip every 12 h Levofloxacin 0.3 g i.v. Drip every 12 h	8 14

**Figure 1:**
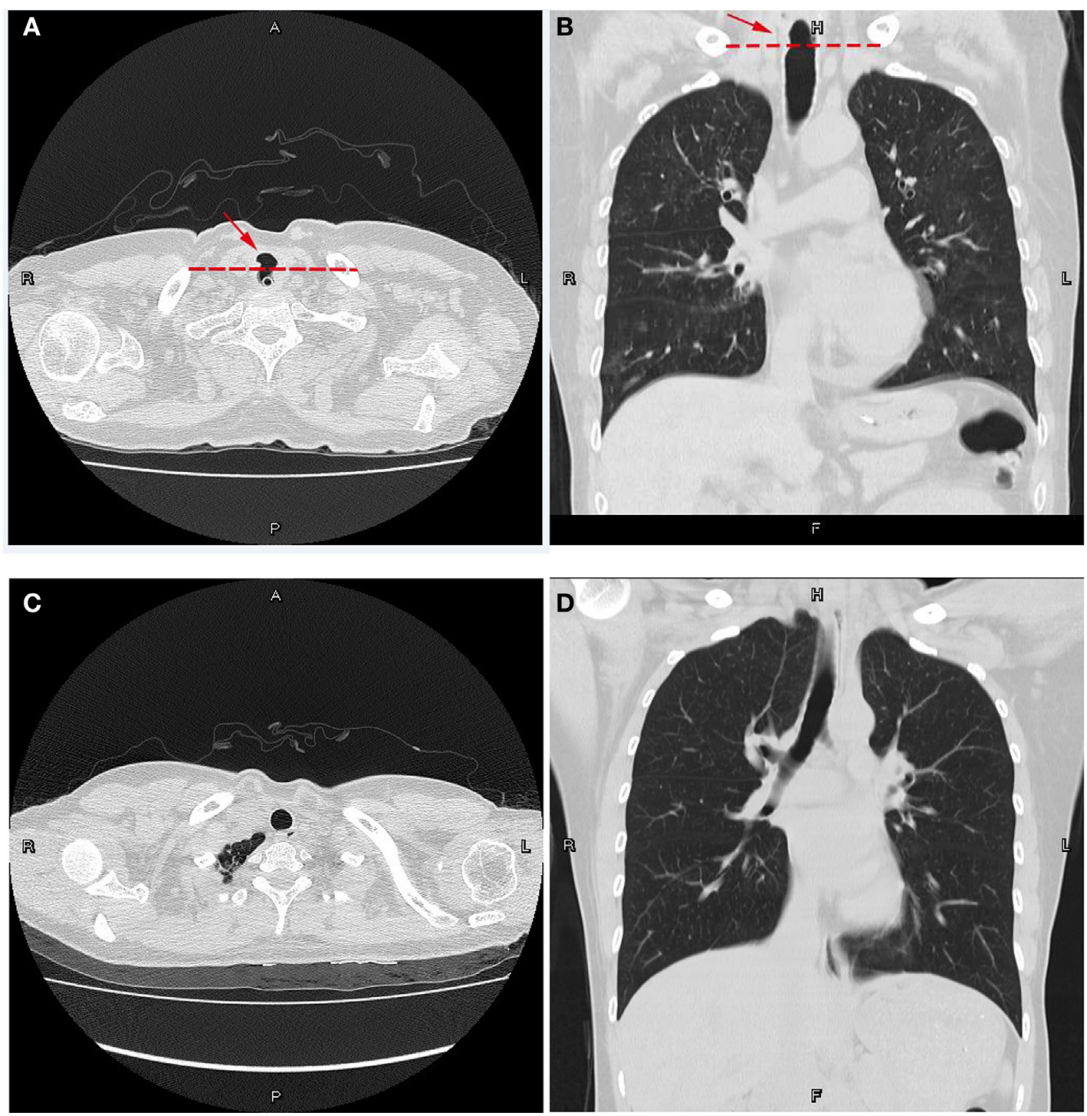
**Computed tomography plain scans of the chest**. **(A,B)** Preoperative views of the patient with a nasogastric tube in place. **(A)** Transverse position. **(B)** Coronal position at the posterior membranous part of the trachea. Note the cavity through the trachea and esophagus. Dotted lines represent the physiological interval. **(C,D)** Postoperative views. **(C)** Transverse position. **(D)** Coronal position. The tracheoesophageal fistula was repaired. The natural physiological interval lies across the trachea and esophagus, remodeling a tracheal cavity and an esophageal lumen.

After 3 weeks of continuous treatment, a chest CT scan revealed a tracheoesophageal fistula, 2 cm in diameter, in the cervical and upper thoracic esophagus (Figures 1A,B). A careful review of a previous CT scan showed that the tracheoesophageal fistula had been present at the time of repeated admission, even last discharge. In an effort to control pulmonary infection, we strengthened the dose of antimicrobial agents, thereby promoting the expulsion of sputum. The patient then stabilized and was transferred to the Department of Thoracic Surgery, where she underwent surgery to repair the fistula (Figures 1C,D). Surgery was successful and the drainage tube, including the nasogastric tube, was removed 1 week later. At this point, she was able to accept a liquid diet without coughing. Three weeks post-operatively, her surgical incision had healed well, and she began a full diet.

## Discussion

Tracheoesophageal fistulas may lead to recurrent and refractory coughing. When the pharyngeal and laryngeal muscles are involved, the clinical manifestations of tracheoesophageal fistulas are similar to those of MG. Both conditions cause coughing while drinking. Consequently, it is easy to misdiagnose these two conditions.

It is important to note that dysphagia is one of the clinical manifestation of MG. Swallowing is a complex activity, involving the coordinated action of the lips, tongue, soft palate, pharynx, and larynx, which are innervated by the facial nerve and cranial nerves IX–XII. In MG patients, the pharyngeal and laryngeal muscles are weak, thus causing difficulty when swallowing; often patients appear to be choking. In addition, swallowing function becomes increasingly worse with sustained swallowing. These symptoms can be relieved with a semiliquid diet. In patients with a tracheoesophageal fistula, however, there is no such characteristic fluctuation. Patients may cough when they have a liquid diet or a semiliquid diet because foreign matter can easily enter the trachea; however, these patients find it easier to swallow these types of diet. When regular treatment does not solve the problem, we should take other possibilities into account.

Airway management is one of the treatments for an acute-phase myasthenic crisis and other diseases that cause dyspnea. Generally, combined endotracheal intubation and tracheostomy is a common and convenient choice (5). The complications associated with this treatment, however, should be taken into account. Tracheal injury can be relatively common during the placement of an artificial airway. Other complications may include tracheal stenosis after tracheostomy, blockage of phlegm following tracheal intubation, tracheoesophageal fistula, and tracheoinnominate artery fistula (6). Tracheoesophageal fistula is an uncommon and easily missed complication. As this condition has clinical characteristics that are similar to those of the primary disease, diagnosticians might fail to consider this rare condition in the differential diagnosis.

In fact, there are several significant risk factors associated with tracheal injuries following the placement of an artificial airway. First, endotracheal intubation may be long lasting. Second, the use of an inappropriately sized tracheal catheter may cause over-loading compression. Third, there is a risk of localized infection and subsequent pathological changes. There are also a range of other factors that could play a role in tracheal injury, including basic diseases (e.g., diabetes, hypoproteinemia), iatrogenic injury, and even the patient’s gender. An earlier report showed that female patients may be more likely to experience iatrogenic tracheal injury because the female tracheal airway is smaller and shorter than that of the male (7–9). In the present case, our female patient, who suffered from diabetes and hypoproteinemia, developed an infection, giving rise to this complex clinical scenario.

## Concluding Remarks

We have described this special case in an attempt to stress that clinical manifestations of the complications of tracheal intubation are varied, and some are insidious. We hope that this report could serve as a reminder for physicians, particularly neurologists, to consider the differences between MG and tracheoesophageal fistula. This will ensure that patients will be given the appropriate attention and treatment without delay.

## Patient Consent

The principle author, Dr. Feng Huiyu, has been the primary doctor for this patient for several years and obtained full consent from the patient to publish this case.

## Author Notes

This study was carried out by the abovementioned authors. The patient provided her written informed consent. The coauthors have agreed that CJX can submit and publish this work on their behalf.

## Author Contributions

The authors declare that they have each made substantial contributions to the conception, acquisition, analysis, and interpretation of the manuscript. All authors have critically revised the manuscript for intellectual content and have given their approval for the final version to be published.

## Conflict of Interest Statement

The authors declare that this research was conducted in the absence of any commercial or financial relationships.

## Abbreviations

q.d., quaque die; q.i.d., quaque in die; MGFA, Myasthenia Gravis Foundation of America.
